# The CXCL13/CXCR5 Immune Axis in Health and Disease—Implications for Intrathecal B Cell Activities in Neuroinflammation

**DOI:** 10.3390/cells11172649

**Published:** 2022-08-25

**Authors:** Christine Harrer, Ferdinand Otto, Richard Friedrich Radlberger, Tobias Moser, Georg Pilz, Peter Wipfler, Andrea Harrer

**Affiliations:** 1 Department of Neurology, Christian Doppler University Hospital, Paracelsus Medical University and Center for Cognitive Neuroscience, 5020 Salzburg, Austria; 2 Clinical Division of Social Psychiatry, Department of Psychiatry and Psychotherapy, Medical University of Vienna, 1090 Vienna, Austria; 3 Department of Dermatology and Allergology, Paracelsus Medical University Salzburg, 5020 Salzburg, Austria

**Keywords:** CXLC13, CXCR5, Tfh cells, B cell, cerebrospinal fluid, ectopic lymphoid structures, neuroinflammation

## Abstract

The chemokine C-X-C- ligand 13 (CXCL13) is a major B cell chemoattractant to B cell follicles in secondary lymphoid organs (SLO) that proposedly recruits B cells to the cerebrospinal fluid (CSF) during neuroinflammation. CXCR5, the cognate receptor of CXCL13, is expressed on B cells and certain T cell subsets, in particular T follicular helper cells (Tfh cells), enabling them to follow CXCL13 gradients towards B cell follicles for spatial proximity, a prerequisite for productive T cell–B cell interaction. Tfh cells are essential contributors to B cell proliferation, differentiation, and high-affinity antibody synthesis and are required for germinal center formation and maintenance. Circulating Tfh cells (cTfh) have been observed in the peripheral blood and CSF. Furthermore, CXCL13/CXCR5-associated immune activities organize and shape adaptive B cell-related immune responses outside of SLO via the formation of ectopic lymphoid structures in inflamed tissues, including the central nervous system (CNS). This review summarizes the recent advances in our understanding of the CXCL13/CXCR5 immune axis and its role in vaccination, autoimmunity, and infection with a special focus on its relevance for intrathecal B cell activities in inflammatory CNS diseases.

## 1. Introduction

An early key role of the C-X-C ligand-13/C-X-C receptor 5 (CXCL13/CXCR5) immune axis during lymphoid organogenesis has been demonstrated in animal models with mice deficient in CXCL13/CXCR5 showing incomplete maturations of lymph nodes [1]. Overexpression of the CXCL13/CXCR5 pathway, in contrast, caused the development of ectopic lymphoid structure(s) (ELS) in non-lymphoid tissue [2].

The chemokine CXCL13, formerly known as B cell attracting chemokine 1 (BCA-1) and B lymphocyte chemoattractant (BLC), is a major B cell chemoattractant and centrally involved in the recruitment of B cells and certain T cell subsets towards B cell follicles in secondary lymphoid organs (SLO) [3,4,5]. CXCL13 exerts its action via the cognate receptor CXCR5 expressed virtually on all B cells and follicular T cells. CXCR5^+^ CD4^+^ T cells migrating in response to CXCL13 towards and within B cell follicles were initially described in tonsils in the early 2000s and termed follicular B helper T (Tfh) cells based on their localization and helper activity for B cell differentiation and antibody production [6,7,8]. Tfh cells are crucially involved in germinal center (GC) formation, extrafollicular and GC antibody responses, affinity maturation, and maintenance of the humoral memory [6,7,9,10,11,12,13]. Thus, CXCL13/CXCR5 immune activities orchestrate both follicle organization and spatial proximity for highly specific T cell–B cell interactions required for the generation of protective antibody responses [6,11,12,13]. Moreover, CXCR5^+^ follicular T cells are not restricted to CD4^+^ Tfh cells but are much more heterogeneous. They include follicular regulatory T cells (Tfr), follicular CD8 T cells, and natural killer T follicular helper cells, which altogether contribute to controlling GC responses [14,15,16,17,18].

Circulating counterparts of Tfh cells (cTfh) have been detected in peripheral blood with increased frequencies in settings of acute and chronic immune activation [19,20,21]. Accumulating evidence revealing heterogeneities in subset differentiation and frequency distributions, and linking the activation states of certain subsets to antigen-specific high-affinity antibody responses, have made them hot topics in infection, vaccination, autoimmunity, and cancer research [19,22,23,24,25,26,27,28,29]. Furthermore, CXCL13/CXCR5-associated immune activities occur in non-lymphoid tissues, where they contribute to organizing and shaping in situ adaptive immune responses via the formation of ELS [30,31] at chronic inflammatory sites during persistent infections, autoimmune diseases, and cancer [2,31,32,33,34,35].

Plenty of evidence furthermore highlights a central role of the CXCL13/CXCR5 axis in the central nervous system (CNS) inflammation [30,36,37,38,39,40,41]. Meningeal ELS were first reported in patients with secondary progressive multiple sclerosis (SPMS) [36,37]. CXCL13 expression has furthermore been detected in active MS lesions [38]. In the cerebrospinal fluid (CSF), CXCL13 elevations occur in a wide spectrum of infectious and immune-mediated neuroinflammatory diseases, associated with CSF pleocytosis, B cell levels, plasmablasts, intrathecal antibody synthesis, and the presence of CXCR5-expressing CD4 T cells [39,41,42,43,44,45,46].

Based on the current understanding of the relevance of the CXLC13/CXCR5 axis in health and disease, we summarize the latest evidence supporting a principal role for CXCL13/CXCR5-associated immune activities in shaping intrathecal B cell responses in acute and active neuroinflammation. Highlights, respectively take home messages, are summarized in Box 1.

Box 1 **Highlights/Take home messages.**

○ CXCL13/CXCR5-associated immune activities orchestrate highly specific B cell–T cell interactions required for protective high-affinity antibody responses in SLO, their balance being of utmost relevance for infection control and prevention of autoimmunity.
○ Soluble and cellular components of the CXCL13/CXCR5 axis are detectable in peripheral blood and CSF and intensely investigated as biomarkers of B cell-related immune activities in the context of infection, vaccination, autoimmunity, and malignancy.
○ CXCL13/CXCR5-associated immune activities organize and shape B cell responses outside of SLO via the formation of ELS in inflamed tissues, including the meninges of the CNS.
○ Meningeal ELS are typically located in cerebral sulci, representing optimal niches for the formation of CXCL13/CXCR5-associated inflammatory foci and B cell-related immune activities, such as intrathecal antibody responses.
○ Pivotal studies from post-mortem SPMS brain tissues highlight meningeal ELS as starting points of intrathecal inflammation across all CNS surfaces and are associated with subpial and deep grey matter damage, emphasizing a key role for CSF as the distributing medium.
○ CSF cell research utilizing sequencing-based single-cell technologies has huge potential in deciphering CXCL13/CXCR5-associated immune cell signatures driving neuroinflammation with relevance for infection control, chronic inflammation, and CNS autoimmunity.

Abbreviations: CNS, central nervous system; CSF, cerebrospinal fluid; CXCL13/CXCR5, C-X-C ligand 13/C-X-C receptor 5; ELS, ectopic lymphoid structures; SLO, secondary lymphoid organs.

## 2. The CXCL13/CXCR5 Immune Axis in the Peripheral Immune System

### 2.1. CXCL13/CXCR5 and Secondary Lymphoid Organs

In SLO, follicular dendritic cells (FDC) and GC-Tfh cells produce CXCL13. This leads to the recruitment of CXCR5^+^ T and B cells towards the B cell zone [3,4,5]. At the T cell–B cell border, follicular bystander B cells license selective pre-Tfh cells to enter the B cell follicle and mature into fully programmed B cell helpers within GC [14,47]. The entire process requires sustained interactions to activate and promote each other. The resultant feedback loops are crucial for GC formation, B cell proliferation, antibody production, and high-affinity maturation, and are therefore essential for protective, pathogen-specific antibody responses and proper regulation of humoral immunity [10,11,13,21].

**Figure 1:**
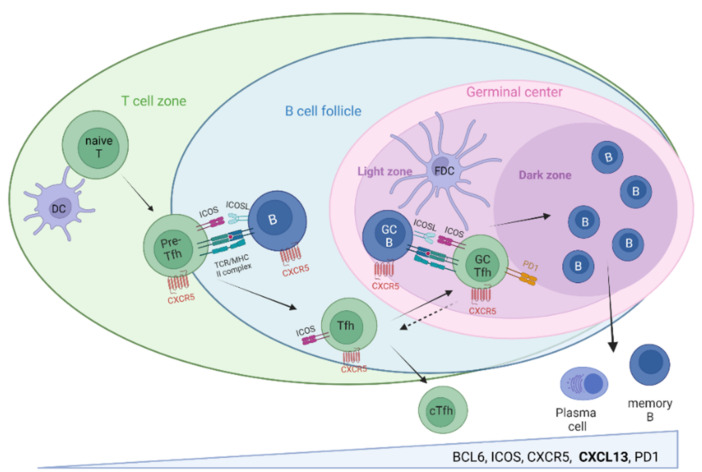
T follicular helper (Tfh) cell and germinal center (GC) development. Initial priming of naive T cells by specific interactions with dendritic cells (DC) leads to the generation of CXCR5+ pre-Tfh cells and their migration along CXCL13 gradients toward B cell follicles. Interactions with cognate B cells at the T cell–B cell border license them to enter the B cell follicle and mature into fully programmed B cell helpers for B cell differentiation, proliferation, and high-affinity maturation within GC. A current model suggests Tfh cells from the T cell–B cell border (solid arrow) rather than GC-Tfh cells (dashed arrow) traveling to the blood, representing as cTfh cells the circulating Tfh cell memory. Created with BioRender.com.

Tfh cells have been known as professional B cell helpers for about two decades [8,48]. The B cell help classically consists of IL-21, IL-10, IL-4, CD40L, and CXCL13 expressions [13]. Fully differentiated GC-Tfh cells are characterized by high levels of lineage-defining transcription factor B cell lymphoma 6 (Bcl6), a high surface expression of CXCR5, inducible T cell co-stimulator (ICOS), and programmed-death receptor 1 (PD1) [9,13,48,49], as demonstrated in Figure 1. Though one of the most numerous effector T cells in lymphoid tissues, GC-Tfh cells are much fewer than GC-B cells and the rate-limiting factor for B cell selection [49]. Thus, they enforce the tight selection of high-affinity B cells required for optimal affinity maturation during GC reactions [50,51,52]. Excessive Tfh cells cause excessive GC formation, GC tolerance breakdown, and a positive selection of aberrant GC-B cells, which produce high-affinity autoreactive antibodies—a proposed key mechanism of systemic autoimmunity [50]. On the other hand, the lack of Tfh cells leads to absent GC formation and compromised B cell development, causing immunodeficiency or insufficient humoral immune responses—a scenario recently observed in the post-mortem tissues of severely infected COVID-19 patients [51].

Importantly, the origin of Tfh cells is not restricted to naïve CD4^+^ T cells imprinted as pre-Tfh cells by interaction with dendritic cells (DC) upon their entry into lymph nodes (LN), but involves other T helper subsets, including CXCR5-expressing Tfh-1, Tfh-2, Tfh-17, and even Tfr and follicular CD8 T cells [21]. Convincing evidence from human tonsils for instance indicates that both Th1 and Th17 cells are able to transdifferentiate into CXCR5-expressing Tfh cells [14,53,54]. The plasticity in Tfh cells has a tremendous impact on the cytokine milieu modulating GC-B cell differentiation and influences the quality and magnitude of antibody responses against various infectious agents, as well as in the case of autoimmunity [55]. Moreover, Tfh-1 cells are more common at the T-B cell border and extrafollicular sites instead of GC, supporting extrafollicular antibody responses during inflammation or following vaccination [13,25,56]. Tfr are CXCR5^+^ subsets of natural regulatory T cells (Tregs), express Tfh-associated genes, including Bcl6, and regulate humoral immunity by suppressing GC reactions [14]. They occur early during infection, accumulate over time, and appear crucial for limiting the development of autoreactive B cells [57]. CXCR5^+^ follicular CD8 T cells, finally, are primarily known for fighting pathogens, such as the Epstein-Barr virus (EBV) and human immunodeficiency virus (HIV), which infect B cell follicles and local Tfh cells [13]. Unexpectedly, CXCR5 + CD8 T cells with reduced cytotoxic potential may alternatively engage as B cell helpers and contribute to shaping antibody responses to viral infections and immunizations [58].

Thus, CXCL13/CXCR5-associated immune activities of SLO involve a gradually discovered arsenal of players contributing to adaptive humoral immunity, their balance being of utmost relevance for infection control and prevention of autoimmunity from top to bottom.

### 2.2. Peripheral Blood

SLO are not hermetically sealed but are connected via lymphatics and circulation allowing traveling and exchange of immune cells. Recent studies have highlighted circulating CXCR5^+^ CD4^+^ T cells (cTfh cells) in peripheral blood as phenotypically and functionally similar counterparts of lymphoid Tfh cells [19,22], which are clonally related [22,59,60,61] but without direct descent from GC-Tfh because the latter rarely egress to circulation [62]. A current model suggests the arising from cTfh cells at the T cell–B cell border and traveling through efferent lymph vessels to the blood [59,61]. Amounting to about 10–20% of CD4^+^ T cells [63], the vast majority are quiescent memory cells expressing the homing molecule CCR7 and are thus capable of entering SLO [64], where they can quickly regain their original phenotypes and participate in GC reactions upon the antigen reencounter [20,49,59,65,66,67,68]. Although they lack the Tfh-lineage defining transcription factor Bcl-6 and the characteristically high ICOS and PD1 surface expressions [49], consensus views them as circulating Tfh cell memory. CXCR5 expression, being the least affected by the compartment switch from SLO to circulation, represents the most reliable marker for tracking cTfh cells [59].

Similar to their lymph node counterparts and based on chemokine receptor expression, transcription factor profile, and cytokine secretion—cTfh are comprised of different subsets, referred to as cTfh-1 (CXCR3 + CCR6, T-bet, IFN-g), cTfh-2 (CXCR3-CCR6-, GATA3, IL-4/IL-5/IL-13) and cTfh-17 (CXCR3-CCR6^+^, RORgt, IL-17/IL-22) [49], differentially regulating B cell responses and capable of antigen-specific B cell help with long-lasting memory [19,35,69]. In vitro, cTfh-2- and cTfh-17 cells efficiently help B cells to proliferate and generate antibodies; cTfh-2 cells promote IgG and IgE secretion, cTfh-17 favor IgG and IgA, whereas cTfh-1 cells appear as non-efficient B cell helpers [19,53,69]. However, the in vivo situation is more complex and cTfh-1 cells may be less important for primary antibody responses; nevertheless, activated vaccine-specific cTfh-1 cells appeared shortly after seasonal influenza vaccination as the predominant cTfh subset and correlated well with the specific antibody response, indicating relevance in the humoral response to antigen recall [25,56].

With blood easily available compared to tissues, investigating cTfh cells and their activation states, frequencies, clonal expansions, and circulating memories quickly emerged as surrogate markers for the GC activity of lymphoid tissues [70] and for monitoring adaptive immune responses to infection and vaccination, including HIV, influenza, and SARS-CoV-2 [23,24,25,56,66,71,72].

It is similarly true for autoimmunity, as increased frequencies of cTfh cells occur in a vast spectrum of both autoantibody-mediated and autoantibody-associated autoimmune diseases, including systemic lupus erythematosus (SLE), rheumatoid arthritis (RA), Sjögren’s syndrome (SS), autoimmune thyroid diseases, myasthenia gravis (MG), neuromyelitis spectrum disorders (NMOSD), type 1 diabetes (T1D), and multiple sclerosis (MS) [26,27,59], frequently associated with a shift towards a higher Th2- plus Th17- to Th1-ratio, higher disease activity, disease severity, and elevated serum CXCL13 levels [9,27,28,29,73,74].

This brings us to CXCL13 as a soluble part of the CXCL13/CXCR5 immune axis, which is, as is the case for cTfh cells, physiologically detectable in blood and increased upon immune activation in the same contexts of infection, vaccination, and autoimmune disorders [34,75,76]. Plasma CXCL13 elevations, contrary to their cellular counterparts, are neither antigen- nor disease-specific. Nevertheless, CXCL13 has been proposed as a blood biomarker for GC activity based on immunization studies, revealing that plasma CXCL13 elevations correlated with activated cTfh cells and the magnitude of antibody responses [77]. Analyzing lymphoid tissue usually is hampered by a lack of tissue availability or thought to interfere with the ongoing immune response. Blood CXCL13, in contrast, is easily available and has been intensely investigated in HIV infection and in response to HIV and influenza vaccination for monitoring vaccination success [77,78,79]. Importantly, whereas antigen-specific antibody production may not be detectable up to six months after primary immunization, CXCL13 elevations usually can be detected after each immunization and provide earlier insights into the immune response and immunization scheme [77].

With regard to autoimmunity, CXCL13 elevations in the blood occur in SLE, RA, SS, MS, and MG, and are proposed biomarkers for disease activity, treatment response, and treatment targets for autoimmune disorders [30,34,80,81,82]. Preclinical therapeutic intervention studies with an anti-CXCL13 monoclonal antibody accordingly showed comparable effectiveness compared to standard treatments in mouse models of RA (collagen-induced arthritis) and MS (experimental autoimmune encephalomyelitis) [82]. Furthermore, CXCL13 and CXCR5 are both overexpressed in B cell chronic lymphocytic leukemia [83]. Increased CXCL13 plasma levels correlate with disease activity and progression, normalize during treatment with Bruton’s tyrosine kinase inhibitor ibrutinib, and increase upon ibrutinib resistance [84].

Thus, the circulating CXCL13/CXCR5-associated components clearly reflect the immune biology of humoral immunity in normal and disease. However, there is one more layer of complexity regarding this immune axis, which is related to the export of humoral immune responses into inflamed tissues via the formation of ELS.

### 2.3. CXCL13/CXCR5 and Non-Lymphoid Tissue

ELS are lymphoid structures outside of SLO and occur at sites of chronic inflammation associated with persistent infection, transplant rejection, autoimmune diseases, and the tumor microenvironment [31,32,85,86]. They lack the fibrous capsule of SLO and form deep within tissue directly exposed to local antigens and the inflammatory milieu [87]. Firmly embedded, they can perfectly participate in local immune responses against chronic infections or cancer for the good, but they can also contribute to developing autoimmunity and drive autoreactive processes for the bad [31,88].

**Figure 2:**
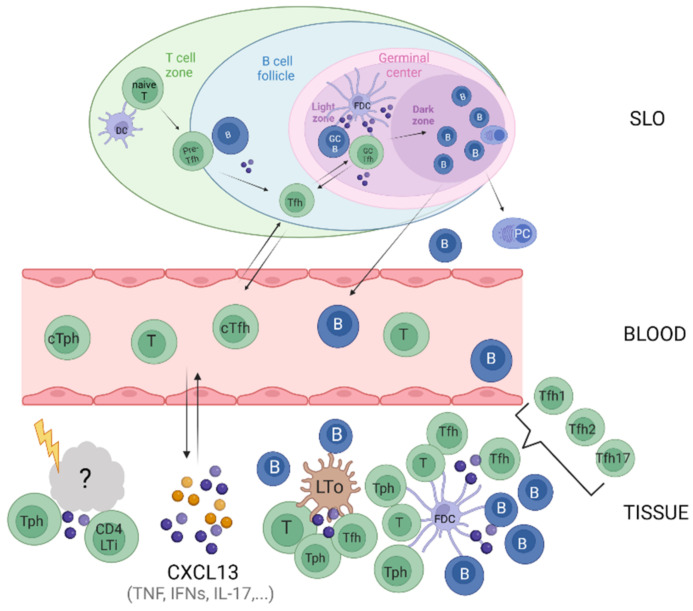
CXCL13/CXCR5-associated immune activities across tissues. Tfh cells are the dominant B cell helper cells in secondary lymphoid tissues (SLO) where Tfh cell–B cell interactions are precisely coordinated. Some Tfh cells, memory B cells, and plasmablasts exit SLO and circulate in the blood. The formation of ELS in inflamed tissues is less resolved. Various cell types, including (but not restricted to) peripheral T helper cells, CD4 lymphoid tissue inducer (Lti) cells, and resident stromal cells assemble in response to sustained inflammation and contribute to the cellular organization of lymphoid aggregates by secreting CXCL13 and other proinflammatory substances, which again lead to further recruitment of immune cells. Resident stromal cells further contribute as tissue organizers (Lto) to the spatial organization of lymphoid aggregates into defined clusters, where follicular dendritic cells (FDC) support the maintenance of B cell activity and their interaction with Tfh cells. Created with BioRender.com.

The primary processes leading to ELS generation are not fully resolved; however, the role of the CXCL13/CXCR5-axis is undisputed. A proposed scenario involves Th17 cells, Tfh cells, and CD4^+^ lymphoid tissue inducer (LTi) cells inducing CXCL13 production in activated stromal cells leading to the attraction of B cells and further Tfh cells and their spatial organization [31,89]. Moreover, they contain Tfh-like cells, termed peripheral T helper (Tph) cells, which lack CXCR5 and Bcl-6 but express CXCL13, IL-21, and CD40L. Recently discovered, these cells are fully competent B cell helpers [90] and a further variation of components associated with the CXCL13/CXCR5 axis (Figure 2).

Permissiveness of the tissue appears critical because ELS preferentially develop in certain tissues, including mucosal sites, salivary glands, synovia, and meninges, have varying degrees of organization, and occur only in some patients [31,89]. Higher degree-structured ELS resemble B cell follicles of SLO, typically contain a central B-cell rich area surrounded by a T cell zone and plasma cells/plasmablasts, a network of CXCL13-producing FDC, stromal mesenchymal cells, and high endothelial venules favoring the migration of lymphocytes [31,91,92]. Such ELS function similar to GC and shape the local adaptive immune response by mediating in situ B cell differentiation, somatic hypermutation, oligoclonal expansion, and in-situ antibody production [13,89,93]. They occur at infection-associated, often mucosal sites of immune defense against bacterial and viral pathogens and in target organs of different autoimmune diseases, including RA, SLE, or SS [31,94,95,96]. However, the majority of ELS are less structured T and B cell aggregates with variable features of the organization but are clearly distinct from diffuse immune cell infiltrates and capable of promoting in situ B cell differentiation and antibody production [90]. The fate of ELS possibly depends on the inflammatory context and structural organization. Some ELS persist until detection in post-mortem tissue analysis, whereas infection-associated ELS reportedly resolve with clearance of the pathogen [89].

## 3. CXCL13/CXCR5 Immune Axis in the CNS

Detection of meningeal lymphoid-like follicles dates back to the early 2000s from studies of post-mortem brain tissues of SPMS patients [97]. Shortly after, CXCL13 expression was reported in active CNS lesions of MS brain tissues [38] and the CSF of patients with Lyme neuroborreliosis (LNB) [97]. Since then, a key role of CXCL13/CXCR5-associated immune activities in the inflamed CNS is evident, the knowledge gained, however, is scarce. In the following, we outline the current knowledge about CNS immunity and integrate lessons learned about the peripheral CXCL13/CXCR5-axis as a framework for translating its relevance to neuroinfection and immune-mediated CNS disorders.

### 3.1. The Meningeal Perspective

The immunological importance of the meninges has long been underestimated. We now know their roles as the gate-keeper for the brain and spinal cord and that they host different immune cell populations of myeloid and lymphoid lineages [98]. Moreover, most CNS immune responses begin in the meninges before gaining access to the parenchyma [98].

Anatomically, the meninges consist of the dura mater, the outermost meningeal layer directly adjacent to the skull containing fenestrated vasculature, a large repertoire of immune cells, including resident immune sentinels, and only recently discovered, lymphatic vessels [99,100]. Directly beneath are the leptomeninges, consisting of the arachnoid and pial layers, and the subarachnoid space, which contains the CSF. The arachnoid mater consists of cells with tight junctions forming the arachnoid barrier, the outer leptomeningeal brain barrier separating the dura mater and entire periphery from the rest of the CNS. Trabeculae and collagen bundles connect the arachnoid to the pia mater, which is permeable for small molecules only, sheathe blood vessels traversing the subarachnoid space, and together with the glia limitans form the innermost barrier to the brain and spinal cord parenchyma [98,99]. The subarachnoid space and pial layer contain resident dendritic cells and long-lived macrophages. As immune sentinels, they present CNS antigens to patrolling CSF immune cells and have a major role in CNS immune surveillance [98].

Sterile and infectious triggers can cause meningeal inflammation, resulting in the degradation of the glia limitans and recruitment and CNS infiltration of further immune cells and, hence, an increase of many neurological disorders [98,101]. This includes the formation of meningeal ELS, which, similar to the periphery, are composed of CXCL13-producing FDC, B cells, plasma cells, T cells, and proliferating B cells, suggesting GC formation [36,37,97].

First described in SPMS in the early 2000s [36,37], these reports of organized niches with prominent CXCL13 expression sustaining CNS inflammation and autoimmunity have directed attention to the role of the meninges in the pathogenesis of MS. Their disease-pathogenic relevance was corroborated by subsequent studies revealing that meningeal ELS occurred in about 40% of SPMS patients and that their presence was associated with early disease onset and severe cortical pathology [37]. Moreover, meningeal B cell follicles occurred close to subpial cortical lesions and their presence was accompanied by pronounced demyelination, microglia activation, and loss of neuritis [37]. These findings were furthermore extended to spinal cord pathology with lymphoid-like structures containing follicular DC networks and dividing B cells reported from spinal leptomeninges in the subgroup of patients with similar findings in their forebrains [102].

Regardless of whether it is more or less structured—meningeal ELS detected in MS brains suggested the meninges as permissive tissue. Sulci reportedly are typical locations for ELS [102], representing optimal niches for circumscribed, self-organizing CXCL13-expressing chronic inflammatory foci potentially facilitating recruitment and exchange of cTfh cells and B cells via CSF or perivascular spaces of meningeal blood vessels.

Though hypothetical, such a scenario is possible since clones of related B cells occur in different pathological sites in the brain, indicating traveling dynamics and active exchange [103]. The latest findings also strongly support this idea. The same group who first reported ELS-like structures in the cortical and spinal leptomeninges proceeded deciphering intrathecal inflammation by turning their attention from the pial surface towards deep grey matter nuclei of post-mortem SPMS brain tissues [104]. Their work culminated in a detailed description of an “ependymal-in” gradient of pathological cell alterations in active lesions of dorsomedial thalamic nuclei, which, similar to subpial grey matter damage, involved a gradient of neuro-axonal loss and microglial activation. Importantly, deep grey matter damage was strongly associated with the presence of cortical meningeal ELS, increased leptomeningeal and CSF inflammation, and elevated B cell-related activity, and possibly indicates a special endo-phenotype of MS [104]. Hence, meningeal ELS are less considered local phenomena but rather may serve as starting points of intrathecal inflammation across all CNS surfaces, at the inside and outside of the brain and spinal cord [104].

**Figure 3:**
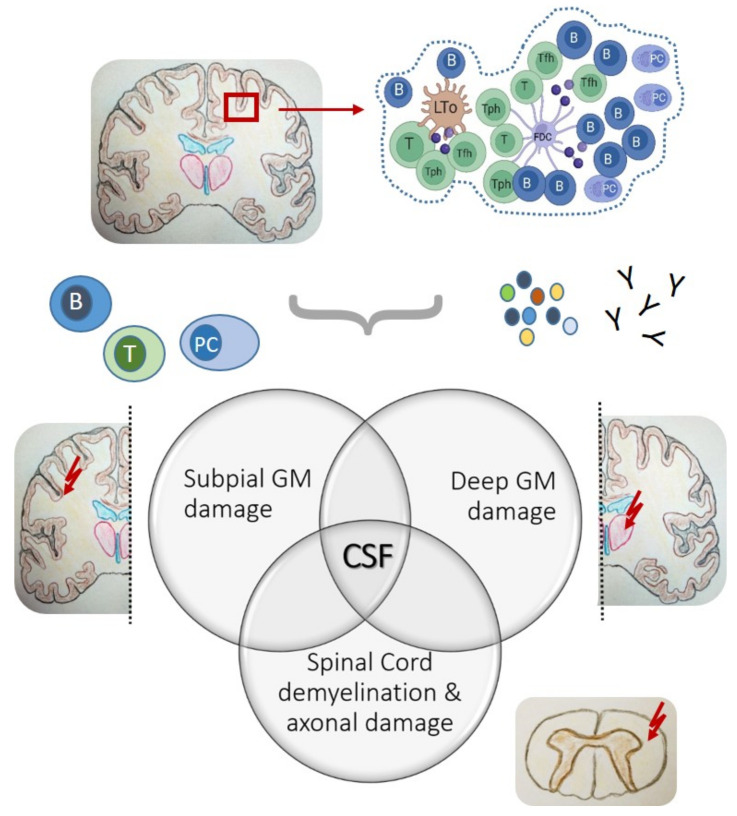
Model of meningeal ELS-associated inflammation according to the current understanding from examining post-mortem CNS tissues of SPMS patients. Meningeal ELS preferentially located in sulci of the forebrain as inflammatory niches of intrathecal B cell–T cell interactions most likely contribute both cellular and soluble factors, which, distributed via CSF, can drive intrathecal inflammation at distant sites, including subpial and deep GM damage and spinal cord demyelination and axonal damage.

However, the question remains as to what the permissiveness in the context of CNS tissue means, in particular, considering the meningeal anatomy with its brain barrier and restriction on immune cell trafficking. Just so much is becoming increasingly clear—as foci of intrathecal adaptive immune activity, meningeal ELS host productive B cell–T cell interactions, and most likely contribute cellular and soluble factors, which, distributed via CSF, can drive intrathecal inflammation and CNS tissue damage at permissive distant sites (Figure 3).

In recent years, the presence and occurrence of meningeal ELS and related structures in the form of B cell-rich immune aggregates have attracted huge interest in a plethora of CNS inflammatory conditions far beyond MS, including neuropsychiatric lupus [105] glioblastoma [106], or focal chronic meningitis with a cocaine-induced midline destructive lesion [107].

Whether meningeal ELS develop or at least transiently exist in acute or chronic neuroinfection as means of CNS immune defense has not been attended to so far. Several lines of evidence point in that direction, such as intrathecal CXCL13 elevations, increased B cell numbers, the occurrence of plasma cells, and intrathecal production of pathogen-specific antibodies—a characteristic constellation, for instance, of LNB [108,109]. ELS-like structures can form in the acute setting as evidenced in a mouse model during acute spinal cord injury [110]. Finally, ELS (their presence or, respectively, persistence) may serve as sites of increased susceptibility for complications such as chronification and post-infectious autoimmunity [111].

Our knowledge about meningeal immunity has dramatically evolved, and even the dura, dural sinuses, dural lymphatics, and the adjacent skull—with skull bone marrow as the myeloid cell reservoir—are proposed to be involved in CNS immune surveillance and as nearby resources of B cells [99,112,113,114,115].

Integrating these CNS border compartments into broader concepts of meningeal immunity may help in discovering unrecognized functional connections between periphery and CNS, including answers regarding the biology of CXCL13/CXCR5-associated adaptive immune activities relevant for intrathecal antibody production.

### 3.2. Cerebrospinal Fluid—Immune Interface between Meninges, Periphery, and CNS

**Figure 4:**
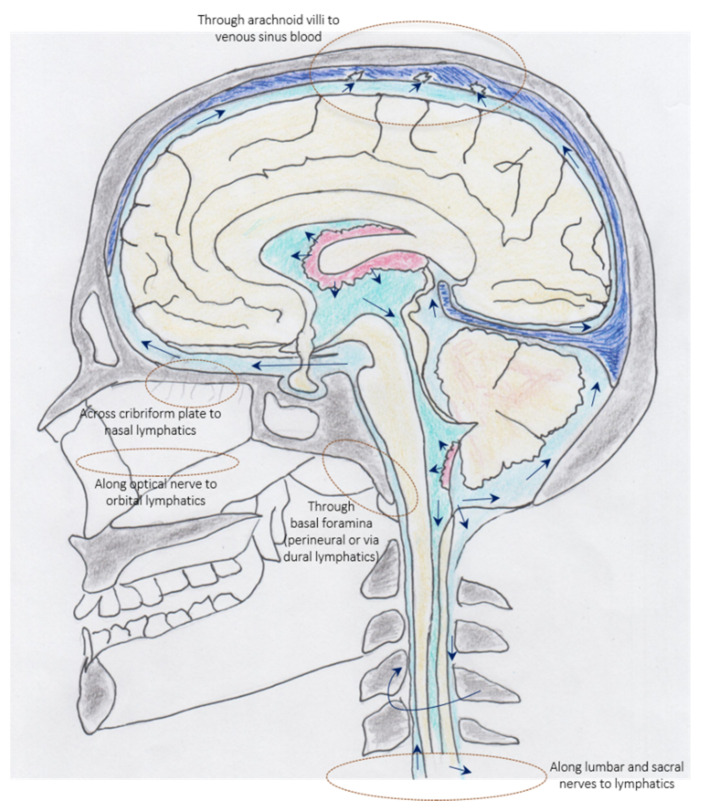
Schematic illustration of exit routes of CSF into blood and lymphatics. Reproduced and modified from Proulx ST. Cerebrospinal fluid outflow: a review of the historical and contemporary evidence for arachnoid villi, perineural routes, and dural lymphatics. Cell Mol Life Sci (2021), https://doi.org/10.1007/s00018-020-03706-5, accessed on 20 June 2022, licensed under a Creative Commons Attribution 4.0 International License, https://creativecommons.org/licenses/by/4.0/, accessed on 20 June 2022. Original scheme by Joachim Birch Milan.

CSF encompasses the brain and spinal cord and provides buoyancy and bidirectional substance distributions to and from parenchyma for nourishment and waste disposal [116]. The bidirectional exchange between CSF and the brain interstitial fluid mainly occurs via diffusion through gap junctions across pial/ependymal linings and the oscillatory flow of CSF in the aqueduct of Sylvius [116]. Although secretion and reabsorption of CSF via cerebral capillaries have been proposed as alternative sources [117], broad consensus is that CSF is ultra-filtrated in the choroid plexus from the blood into the ventricular system, passes from the ventricular system to the subarachnoid space, flows down to the distal spinal cord, and mostly back into the cranium [116,118]. It exits by drainage through arachnoid granulations into venous sinus blood, across the cribriform plate, the basal foramina, optical nerve, and via spinal nerve roots into the lymphatics (Figure 4), with the strongest supporting evidence proposed for the route across the cribriform plate [116,118,119].

Whatever way, CSF carries and drains CNS antigens, which can accumulate in the deep cervical LNs where they may help educate self-tolerance to lymphocytes licensed for patrolling the CNS, and as recently proposed, dural stromal niches [98,99,114].

Normal CSF contains extremely few cells, a special selection of blood-derived immune cells, primarily composed of central memory CD4 T cells and monocytes, which are capable of crossing the protective brain barriers of the choroid plexus, leptomeninges, and post-capillary venules of the blood-brain barrier [90,99,120,121,122,123,124]. CSF also contains cTfh cells occurring at frequencies similar to peripheral blood, whereas B cells and plasma cells are usually absent [38,121,125].

According to the current understanding of normal CNS immune surveillance, CSF immune cells patrol the subarachnoid, ventricular, and perivascular CSF spaces, encounter resident CNS antigens presenting immune sentinels, and return to the circulation or lymphatics [98,120,126,127,128].

CSF pleocytosis, which involves increased recruitment of immune cells from peripheral blood to CSF and local proliferation, is a hallmark finding of infectious and immune-mediated CNS disorders, and frequently involves the appearance of B cells and plasma cells [129,130]. Their relevance for immune defense is supported by the detection of intrathecal antibody production against neurotropic pathogens, such as HSV, VZV, rubella, measles, tickborne encephalitis, or Lyme neuroborreliosis [109]. Their relevance in immune mediated-disease, such as MS, MS-related disorders, and autoimmune encephalitis is supported by the detection of intrathecal antibody production against ubiquitous intracellular self-protein and specific CNS antigens, including Aquaporin-4, Myelin Oligodendrocyte Glycoprotein (MOG), or N-methyl-D-aspartate receptor (NMDAR) [109,131].

Moreover, from MS research, it is well established that clonally related B cells exist in blood and CSF, which undergo further diversification within their respective compartments, participate in the bidirectional exchange between compartments, and travel toward other sub-compartments of the CNS (meninges or parenchyma) [125,131].

The driving force behind their recruitment to CSF, local antibody production, and further diversification involving somatic hypermutation is less well understood. At this point, the idea of a functional intrathecal CXCL13/CXCR5-axis appears worth considering, because CXCL13, the chemokine most strongly implicated in B cell migration, is frequently elevated in the inflammatory CSF but below the detection limit or detectable only at trace amounts in normal CSF [40,75,121].

At about the time of early reports in CNS tissues, CXCL13 was detected in the CSF of patients with LNB, revealing relevance in neuroinfection [132]. Based on striking high elevations, several groups dedicated serious efforts to establish CXCL13 as a diagnostic biomarker of LNB [39,43,133,134]. However, consensus has not been achieved because CXCL13 was found elevated in a broad spectrum of viral and bacterial infections, and immune-mediated and neoplastic CNS disorders [40,135], including varicella zoster virus facial palsy [136], neurosyphilis [137], MS [138,139], autoimmune encephalitis [140], and CNS lymphoma [141]. Moreover, in most of these inflammatory conditions, the occurrence and course of CXCL13 elevations have been attributed to prognostic/predictive values and were informative regarding treatment response [139,140,141].

Regarding the associated intrathecal inflammatory milieu evidence accumulated, that intrathecal CXCL13 elevations strongly correlated with CSF pleocytosis, but also with B cell numbers, intrathecal immunoglobulin synthesis, and according to recent findings, with frequencies of CXCR5^+^ CD4^+^ T cells [44,46,122,123]. A role of CXCL13 in the recruitment of B cells to CSF has already been suggested earlier [44]. Apparently, there is more to it, as both B cells and their professional helper T cells co-occurred with CXCL13 elevations in the inflamed CSF [75]. This notion is corroborated by the first studies proposing intrathecal cTfh cells relevant for driving B cell expansion and differentiation in MS, and for the affinity maturation of pathogen-specific antibodies in virus-associated neuroinflammatory diseases [45,111].

The question is where and how. Immune cells being flushed with CSF upon entry might not allow productive B cell–T cell interactions; however, they have the opportunity to travel toward remote sites and reach niches for acute or sustained neuroinflammation and/or the spread of grey matter damage [104,142].

The situation could also be the other way around, meaning that CXCL13 does not recruit its cognate B and cTfh cells to CSF, but that all three, more or less accidentally, originate from (more or less) remote inflammatory meningeal or parenchymal brain or spinal cord sites. This view again is supported by the microglia as a major source of CXCL13 as observed in mice infected with a neuroadapted Sindbis virus [143]. Perhaps both scenarios exist in parallel, interacting with and sustaining each other.

Notably, such a scenario perfectly complies with the current knowledge about intrathecal sources of CXCL13, which are heterogeneous and include inflammatory microglia in actively demyelinating CNS lesions, FDC, and CXCL13-producing Tfh and Tfh-like cells in meningeal tertiary lymphoid structures (TLS), perivascular follicular stromal cells in CNS lymphoma, and intrathecal macrophages in LNB [36,37,38,40,144]. Moreover, the extent of CXCL13 elevations is highly variable and seemingly dependent on the underlying immune pathogenic condition, location, disease course, and response to therapy [40,42]. The highest levels occur in spirochetal CNS infections, such as LNB, neurosyphilis, and CNS lymphoma [43,137,144], whereas lower to intermediate levels predominate in other bacterial and viral infections and immune-mediated and neoplastic CNS disorders [39,40,44,135].

Which scenario is currently closer to reality is of secondary importance. CSF is a highly relevant immune interface and, in contrast to CNS tissue, easily available. Thus, it represents a most promising source for deciphering the intrathecal immune landscape of CXCL13/CXCR5-associated effectors, their subset compositions, and activation states similar to what is known from blood. Accordingly, the cytokine milieu provided by predominating Tfh-2, Tfh-17, or Tfh-1 subsets, or combinations thereof, may inform about B cell differentiation and the quality of intrathecal antibody responses reflective of an infectious or immune-mediated etiology. Furthermore, the presence of Tfr and CXCR5^+^ CD8 T cells may indicate competence for immune control or whether additional help in local antibody synthesis is required.

## 4. Conclusions

CXCL13 potentially recruits not only B cells but also Tfh-like cells to the inflamed CNS, which accumulate and organize in ELS and ELS-like structures adjacent to inner and outer surfaces across the brain and spinal cord. From within these niches, their productive interaction may be the missing link to the poorly understood phenomenon of intrathecal antibody synthesis and B cell-related adaptive immune responses. Most of our knowledge about meningeal ELS currently stems from detailed examinations of post-mortem SPMS brain tissues. However, increasing evidence from other immune-mediated and malignant CNS diseases emphasizes a broad presence of these sites of structured CXCL13/CXCR5-dependent immune activities. Given the frequently observed CXCL13 elevations in inflammatory CSF, a role in neuroinfection with all its implications in cases of severe disease courses is highly plausible. Animal models will definitely be major sources for increasing our understanding of the dynamics of the formation and resolution of meningeal ELS. Regarding human CNS disease, advancing CSF research appears the most promising. At the very front are modern single-cell technologies and bioinformatics solutions allowing the combined sequencing-based analysis of surface proteins, RNA expressions, and T and B cell receptor repertoires of single cells. Using such potent approaches for identifying circulating specific immune signatures of intrathecal ELS activity will open new dimensions in deciphering CXCL13/CXCR5-associated immune activities as drivers of neuroinflammation and possible therapeutic targets with relevance for infection control, chronic inflammation, and CNS autoimmunity.
